# Novel *PLCG2* Mutation in a Patient With APLAID and Cutis Laxa

**DOI:** 10.3389/fimmu.2018.02863

**Published:** 2018-12-14

**Authors:** João Farela Neves, Rainer Doffinger, Gabriela Barcena-Morales, Catarina Martins, Olivier Papapietro, Vincent Plagnol, James Curtis, Marta Martins, Dinakantha Kumararatne, Ana Isabel Cordeiro, Conceição Neves, Luis Miguel Borrego, Matilda Katan, Sergey Nejentsev

**Affiliations:** ^1^Primary Immunodeficiencies Unit, Hospital Dona Estefânia—CHLC, EPE, Lisbon, Portugal; ^2^CEDOC, Chronic Diseases Research Center, NOVA Medical School, Lisbon, Portugal; ^3^Department of Clinical Biochemistry and Immunology, Addenbrooke's Hospital, Cambridge, United Kingdom; ^4^Laboratorio de Inmunologia, Facultad de Estudios Superiores Cuautitlán, Universidad Nacional Autónoma de México, Mexico City, Mexico; ^5^Department of Medicine, University of Cambridge, Cambridge, United Kingdom; ^6^University College London Genetics Institute, University College London, London, United Kingdom; ^7^Faculty of Medicine, University of Lisbon, Lisbon, Portugal; ^8^Immunoallergy Department, Hospital CUF Descobertas, Lisbon, Portugal; ^9^Structural and Molecular Biology, Division of Biosciences, University College London, London, United Kingdom; ^10^Department of Molecular Cell Biology and Immunology, Amsterdam University Medical Centers, Amsterdam, Netherlands

**Keywords:** APLAID, PLCγ2, cutis laxa, sensorineural deafness, IL-10, IL-1b, auto-inflammatory syndromes

## Abstract

**Background:** The auto-inflammation and phospholipase Cγ2 (PLCγ2)-associated antibody deficiency and immune dysregulation (APLAID) syndrome is a rare primary immunodeficiency caused by a gain-of-function mutation S707Y in the *PLCG2* gene previously described in two patients from one family. The APLAID patients presented with early-onset blistering skin lesions, posterior uveitis, inflammatory bowel disease (IBD) and recurrent sinopulmonary infections caused by a humoral defect, but lacked circulating autoantibodies and had no cold-induced urticaria, contrary to the patients with the related PLAID syndrome.

**Case:** We describe a new APLAID patient who presented with vesiculopustular rash in the 1st weeks of life, followed by IBD, posterior uveitis, recurrent chest infections, interstitial pneumonitis, and also had sensorineural deafness and cutis laxa. Her disease has been refractory to most treatments, including IL1 blockers and a trial with ruxolitinib has been attempted.

**Results:** In this patient, we found a unique *de novo* heterozygous missense L848P mutation in the *PLCG2* gene, predicted to affect the PLCγ2 structure. Similarly to S707Y, the L848P mutation led to the increased basal and EGF-stimulated PLCγ2 activity *in vitro*. Whole blood assays showed reduced production of IFN-γ and IL-17 in response to polyclonal T-cell stimulation and reduced production of IL-10 and IL-1β after LPS stimulation. Reduced IL-1β levels and the lack of clinical response to treatment with IL-1 blockers argue against NLRP3 inflammasome hyperactivation being the main mechanism mediating the APLAID pathogenesis.

**Conclusion:** Our findings indicate that L848P is novel a gain-of-function mutation that leads to PLCγ2 activation and suggest cutis laxa as a possible clinical manifestations of the APLAID syndrome.

## Introduction

The strict control of inflammation is one of the most important functions of the immune system (1). While the majority of the autoimmune inflammatory diseases are mediated by effectors of the adaptive immune system, auto-inflammatory syndromes are caused by an aberrant activation of the innate immune cells (1–4). These disorders are characterized by systemic inflammation and diverse organ-specific manifestations, such as arthritis, aseptic meningitis, deafness, or urticaria (4, 5). More than 20 years ago, mutations in the *MEFV* and *TNFRSF1A* genes were shown to cause familial Mediterranean fever and Tumor necrosis factor receptor-associated periodic syndrome (TRAPS) (6). Since then, inherited deficiencies in the components of the innate immune system that control inflammation have been identified, e.g., cryopyrin-associated periodic syndrome (CAPS) and Auto-inflammation with infantile enterocolitis (AIFEC) caused by gain-of-function mutations in NLRP3 and NLRC4 that lead to the release of pro-inflammatory cytokines (such as IL-1β) (7–9). These findings expanded the knowledge about mechanisms mediating auto-inflammation and provided scientific basis for the targeted treatment of such disorders (4–7).

Phosphatidylinositol and its derivatives are essential regulators of the human immune cell functions. Phospholipase C (PLC) is a family of enzymes that catalyze hydrolysis of phosphatidylinositol 4,5-bisphosphate, producing second messenger molecules diacylglycerol (DAG) and inositol 1,4,5-trisphosphate (IP_3_). IP_3_ induces release of calcium from endoplasmic reticulum to the cytoplasm, while DAG can activate cell signaling proteins, e.g., protein kinase C. PLCγ is structurally distinct from other PLC enzymes and has two isoforms. PLCγ1 is ubiquitously expressed and acts downstream of growth factor receptors, while PLCγ2 is found mainly in hematopoietic cells, where it is activated by tyrosine kinases recruited to immune cell receptors, e.g., BCR and Fc receptors or, as subsequently shown, also by Rac GTPase (10–15).

Recently, 27 patients presenting with cold-induced urticaria, recurrent sinopulmonary infections, antibody deficiency, and autoimmunity, were found to have heterozygous in-frame deletions affecting the *PLCG2* gene, resulting in constitutive activation of PLCγ2. This syndrome was designated the PLCγ2-associated antibody deficiency and immune dysregulation (PLAID) (10). Thereafter, the auto-inflammation and PLCγ2-associated antibody deficiency and immune dysregulation (APLAID) syndrome was described in two patients from another family (11). APLAID patients presented with early-onset blistering skin lesions, eye inflammation with ocular hypertension, inflammatory bowel disease (IBD), arthralgia, and recurrent sinopulmonary infections caused by a humoral defect, but lacked circulating autoantibodies and had no cold-induced urticaria. This distinctive phenotype was caused by a missense mutation S707Y in the *PLCG2* gene that disrupted an auto-inhibitory cSH2 domain, causing increased production of intracellular IP_3_ and calcium release resulting in hyperactivation of the PLCγ2 signaling pathway (11). Contrary to the PLAID mutants, this mutation does not lead to constitutive enzymatic activity, requiring upstream signaling events for its activation.

Here, we report a new patient with APLAID caused by a unique activating mutation in PLCγ2 that presented with novel manifestations, including cutis laxa and sensorineural deafness.

## Materials and Methods

### Ethics

All materials were obtained with informed consent in accordance with the Declaration of Helsinki and with approval from the ethics committees in Portugal (131/2014) and UK (15/WS/0019). Written informed consent for publication of this case report was obtained from the parents of the patient.

### Flow Cytometry

For the evaluation of lymphocyte subsets, peripheral blood was analyzed by Flow Cytometry in a 4-color BD FACS Calibur (BD, San Jose, California, USA), using the BD IMK kit with Trucount tubes (BD Biosciences) according to the manufacturer's instructions. An additional panel of monoclonal antibodies (including CD45RA, CD45RO, CD62L, and HLA-DR) was also performed for further characterization of T-cell differentiation, using a lyse-wash protocol. Finally, the IOTest® Beta Mark kit (Immunotech SAS, a Beckman Coulter Company, Marseille, France) was also used for the characterization of the TCR Vβ repertoire.

Flow cytometry data analysis was performed using Multiset and CellQuestPro software (BD Biosciences).

### Proliferation Assays

Proliferative capacity was performed with a thymidine incorporation assay. In brief, peripheral blood mononuclear cells (PBMCs) were incubated with mitogens (PHA, final concentration and PMA+ionomycin) for 3–4 days, and with antigens (PPD, C. albicans antigen and Tetanus toxoid) for 7–8 days, at 37°C, in a 5% CO_2_ atmosphere. The cells were labeled with tritiated thymidine (3H-thymidine) (Perkin Elmer, Boston, MA, USA) in the last 18 h of incubation. Cells were then harvested and transferred to a Filtermat A filter (Perkin Elmer), and a Meltilex scintillation sheet (Perkin Elmer) was applied after the Filtermat was dried. The radioactivity in DNA recovered from the cells was read in a MicroBeta counter (Perkin Elmer). Results were presented as stimulation indexes, obtained from the ratios of cpm from stimulated and unstimulated cells (incubated in parallel).

### Antibody Response Evaluation

Immunoglobulins were quantified in serum. Immunoturbidimetria was used to quantify IgG, IgA, and IgM. As for specific responses, antibodies for Diphtheria, Tetanus, and Pertussis were evaluated by ELISA.

### Whole-Blood Cytokine Production Assays

Whole blood was diluted 1:5 in RPMI into 96-well F plates (Corning) and activated using the following stimulants Phytohemagglutinin (PHA; 10 μg/ml; Sigma-Aldrich) PMA/IONO, LPS (1 μg/ml, List Biochemicals), Pam2CSK4 (Invivogen, 1 micg/ml), Pam3CSK4 (Invivogen, 1 micg/ml), Flagellin (Invivogen, 1 micg/ml), CL-097 (Invivogen, × micg/ml). Supernatants were taken at 24 h. Cytokines were measured using multiplexed particle based bead array (IFNγ, TNFα, IL-17,IL-1b, IL-10, IL-6, R+D Systems Fluorokinemap) on a Luminex analyser (Bio-Plex, Bio-Rad, UK). Data were statistically analyzed by the two-tailed Mann-Whitney test using Prism 6 (GraphPad Software).

### Exome Sequencing and Bioinformatics Analysis

We isolated DNA samples from blood or peripheral blood mononuclear cells (PBMCs). Library preparation, exome capture, and sequencing have been done according to the manufacturers' instructions. For exome target enrichment Agilent SureSelect 50 Mb kit was used. Sequencing was done using Illumina HiSeq 2000 with 94 bp paired-end reads. FASTQ files were aligned to the hg19 reference sequence using Novoalign version 2.07.19, including hard and soft clipping, quality calibration and adapter trimming. Duplicate reads were excluded using the PICARD tool MarkDuplicates. Calling was performed using SAMtools v0.18 and single sample calling. The resulting calls were annotated with the software ANNOVAR. Candidate variants were filtered based on function: loss-of-function, non-synonymous or potential splicing altering variants (defined as being with 5 bp of the actual splice site) and frequency.

### Cell Culture and Transfections

Plasmids for the expression of full-length human pTRIEX-PLCg2 constructs in mammalian cells have been described previously (11).

QuikChange PCR mutagenesis (Stratagene) was used to introduce a point mutation in the PLCg2 WT variant. All mutants were fully sequenced to verify the fidelity of PCR. COS7cells were maintained at 37°C in a humidified atmosphere of 95% air and 5% CO2

In Dulbecco's modified Eagle's medium (DMEM) (Invitrogen) supplemented with 10% (v/v) fetal bovine serum (Invitrogen) and 2.5 mM glutamine. Prior to transfection cells were seeded into 6-well plates at a density of 2.5 × 10^5^ cells/well and grown for 16 h in 2 ml/well of the same medium. For transfection, 1.0 ug of PLCg2 DNA was mixed with 1 μl of PlusReagent^TM^ and 7 μl of Lipofectamine^TM^ (Invitrogen) and added to the cells in 0.8 ml of DMEM without serum. The cells were incubated for 3.5 h at 37°C, 5% CO2 before the transfection mixture was removed and replaced with DMEM-containing serum.

### Analysis of Inositol Phosphate Formation in Intact COS7 Cells

Inositol Phosphate formation was assessed as described in Everett et al. (13). Briefly, 24 h after transfection, the cells were washed twice with inositol-free DMEM without serum and incubated for 24 h in 1.5 ml of the same medium supplemented with 0.25% fatty acid free bovine serum albumin (Sigma) and 1.5 uCi/ml myo-[^2−3^H]inositol (MP Biomedicals). After a further 24 h, the cells were incubated in 1.2 ml of inositol-free DMEM without serum containing 20 mM LiCl with or without stimulation with 100 ng/ml EGF (Calbiochem). The cells were lysed by addition of 1.2 ml of 4.5% perchloric acid. After incubating the samples on ice for 30 min, they were centrifuged for 20 min at 3700 × g. Supernatants and pellets were separated. The supernatants were neutralized by addition of 3 ml of 0.5 M potassium hydroxide/9 mM sodium tetraborate and centrifuged for a further 20 min at 3700 × g. Supernatants were loaded onto AG1-X8 200–400 columns (Bio-Rad) that had been converted to the formate form by addition of 2 M ammonium formate/0.1 M formic acid and equilibrated with water. The columns were washed three times with 5 ml of 60 mM ammonium formate/5 mM sodium tetraborate, and inositol phosphates were eluted with 5 ml of 1.2 M ammonium formate/ 0.1 M formic acid. Five milliliter Ultima-Flo scintillation fluid (PerkinElmer Life Sciences) was added to the eluates and the radioactivity quantified by liquid scintillation counting. The values represent total inositol phosphates. The pellets from the first centrifugation were resuspended in 100 μl of water and 375 μl of chloroform/methanol/HCl (200:100:15) was added. The samples were vortexed, and an additional 125 μl of chloroform and 125 μl of 0.1 M HCl were added.

After further vortexing, the samples were centrifuged at 700 × g for 10 min. Ten microliter of the lower phase were placed in a scintillation vial with 3 ml of Ultima-Flo scintillation fluid and the radioactivity quantified by liquid scintillation counting. The obtained values correspond to radioactivity in inositol lipids. PLC activity is expressed as the total inositol phosphates formed relative to the amount of [^3^H]myo-inositol in the phospholipid pool. Because the differences in steady state labeling of inositol lipids are small (within 20%), this normalized PLC activity corresponds closely to PLC values expressed as total inositol phosphates; however, the error bars between the duplicates are generally smaller.

### Case Report

**Figure 1:**
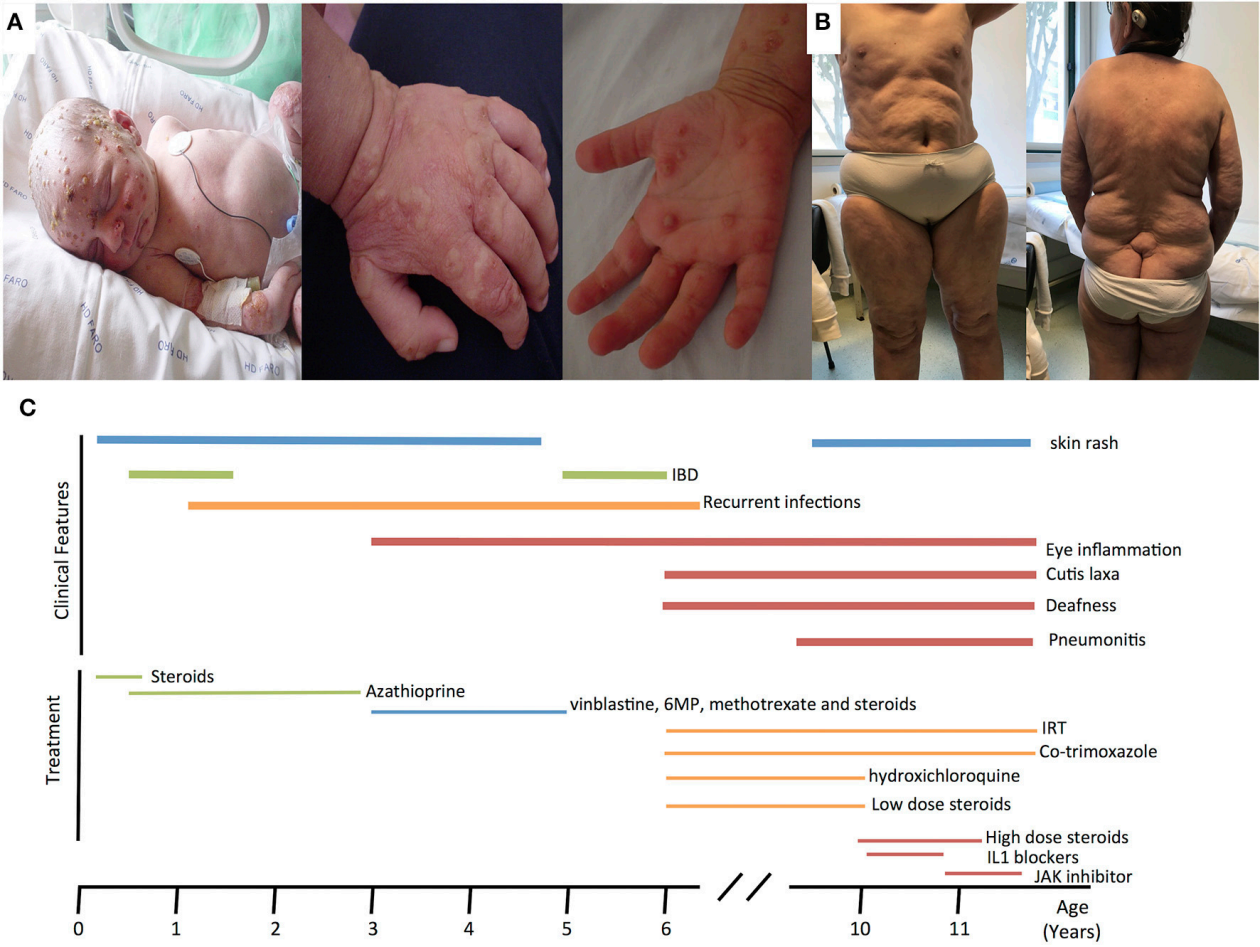
**(A)** Neonatal pustulosis. **(B)** Cutis laxa. **(C)** Timeline for the clinical manifestations and treatments of the patient. IRT, Immunoglobulin replacement therapy.

**Figure 2:**
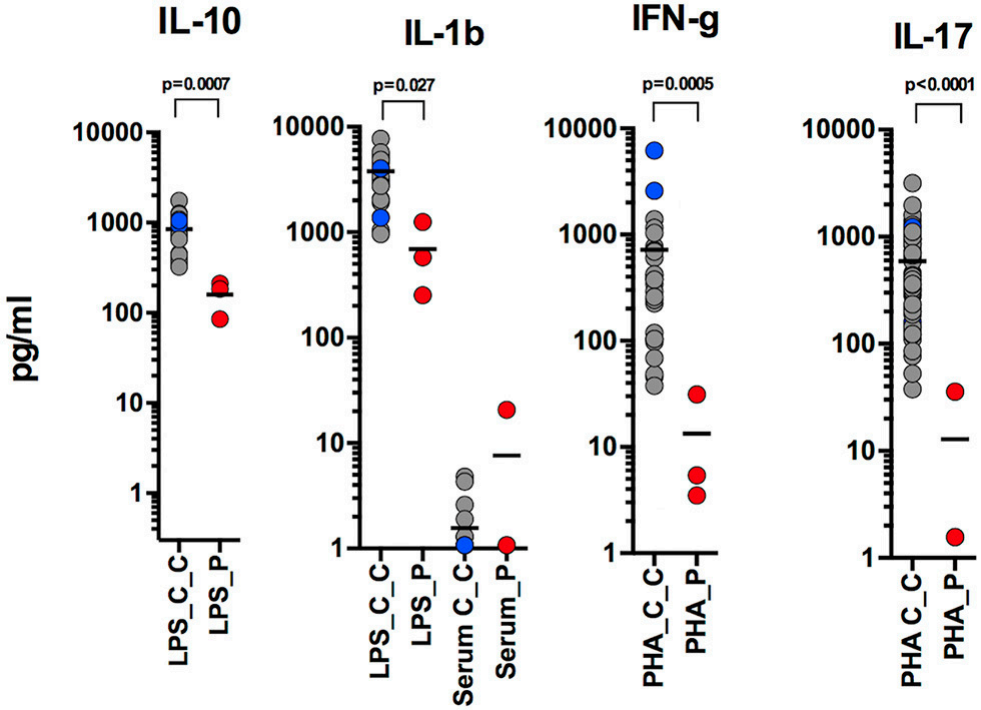
Reduced production of IL-10 and IL-1β after LPS stimulation; reduced production of IFN-γ and IL-17 in response to polyclonal T-cell stimulation (performed at 9, 10, and 11 years-old).

Here, we report an 11 years-old girl, second daughter of healthy non-consanguineous parents of Portuguese origin. She presented with very severe vesiculo-pustular rash in the 1st week of life (Figure 1A). Biopsy showed dermis infiltration by CD68+CD163+S100–CD1a–histiocytes and a presumptive diagnosis of juvenile xanthogranuloma was made. At the age of 2 months, she started bloody diarrhea and early-onset IBD was diagnosed based on the endoscopic and histologic findings. The bowel disease improved after treatment with prednisolone and maintenance with azathioprine. Nevertheless, she continued to have recurrent episodes of blistering skin rash and presented recurrent chest infections. At the age of 3 years she started to have recurrent eye inflammation that was associated with ocular hypertension. Systemic xanthogranuloma was suspected and she was treated with vinblastine, 6-mercaptopurine, methotrexate, and steroids. Her symptoms did not resolve and she had more frequent infections, including acute otitis media, recurrent chronic sinusitis, recurrent pneumonia, and on one occasion, pulmonary *Aspergillus* infection that responded to itraconazole. Cutis laxa was evident since she was 6 years-old (Figure 1B) and progressive sensorineural deafness was diagnosed at the age of 7, requiring a hearing-assistive device. Immunological analysis at that age revealed low IgM and IgA, absent responses to protein antigens and low B cells, with almost absent class-switched memory B cells. T-cell proliferation responses were preserved. No auto-antibodies were found (Table 1). Whole blood stimulation assays showed a strongly reduced production of IFN-γ and IL-17 in response to polyclonal T-cell stimulation and reduced production of IL-10 and IL-1β after LPS stimulation (Figure 2). However, there was a significant innate induction of IL-6 and TNFα in response to various TLR agonists including LPS, PAM 2/3, Flagellin, and CL-097 (Supplementary Figure 1 and Supplementary Table 1 ). She started prophylactic co-trimoxazole, immunoglobulin replacement, as well as low dose steroids (5 mg every other day) and hydroxychloroquine (Figure 1C), which led to substantial improvement. Nevertheless, since the age of 10 years she started to have several relapses of eye inflammation, skin rash, and pulmonary inflammation (interstitial pneumonitis) that improved only partially with high-dose steroids. Anakinra (IL1-receptor antagonist) and canakinumab (IL1β monoclonal antibody) were tried without any effect. In view of the excellent results of the use of JAK inhibitors in auto-inflammatory diseases (16), and given that PLCγ2 requires upstream signaling events for its activation, we have very recently decided to treat the patient with ruxolitinib as a bridge to hematopoietic stem cell transplantation.

**Table 1:** Immunological assessment of the patient.

**Immunology**	**6 Years-old**	**10 Years-old**	**Reference value**
Absolute lymphocyte count ( × 10^9^/L)	3,600	3,350	2,500–5,500
CD3+(cells/μL)	1,685	1,250	1,200–3,000
CD4+(cells/μL)	985	634	650–1,500
CD4+CD45RA+ (%)	65%	59%	53–86%
CD8+(cells/μL)	640	529	370–1,100
CD8+CD45RA+ (%)	83%	79%	42–82%
CD16+/56+ (cells/μL)	100	143	100–480
CD19+(cells/μL)	**↓ 55**	**↓ 143**	270–860
CD19+CD27-IgD+IgM+ (%)	**↑ 90%**	**↑95%**	47–70%
CD19+CD27+ (%)	**↓ 6%**	**↓ 3%**	7–24%
CD19+CD27+IgD- (switch)	**↓ 0.5%**	**↓ 0**	2.7–12.5%
CD19+CD38+IgM^high^ (transitional)	**↑ 50%**	**↑64%**	
IgG (g/L)	**3,563**	**10.5 (on IRT)**	5.3–10.7
IgM (g/L)	**0**	**0**	0.46–1.9
IgA (g/L)	**0**	**0**	0–1.5
Anti-diphteria	**0**	**2.4 (on IRT)**	Protective >0.5
Anti-tetanus	**0**	**1.5 (on IRT)**	Protective >0.5
Proliferative responses to PHA (CPM × 10^3^) (stimulation index)	25,621 ± 1,762 133	Not performed	Control 20,076 ± 3,179 45.8
Proliferative responses to PMA+I (CPM × 10^3^) (stimulation index)	21,665 + 2,320 75	Not performed	Control 13,069 ± 1,645 47.7

**Figure 3:**
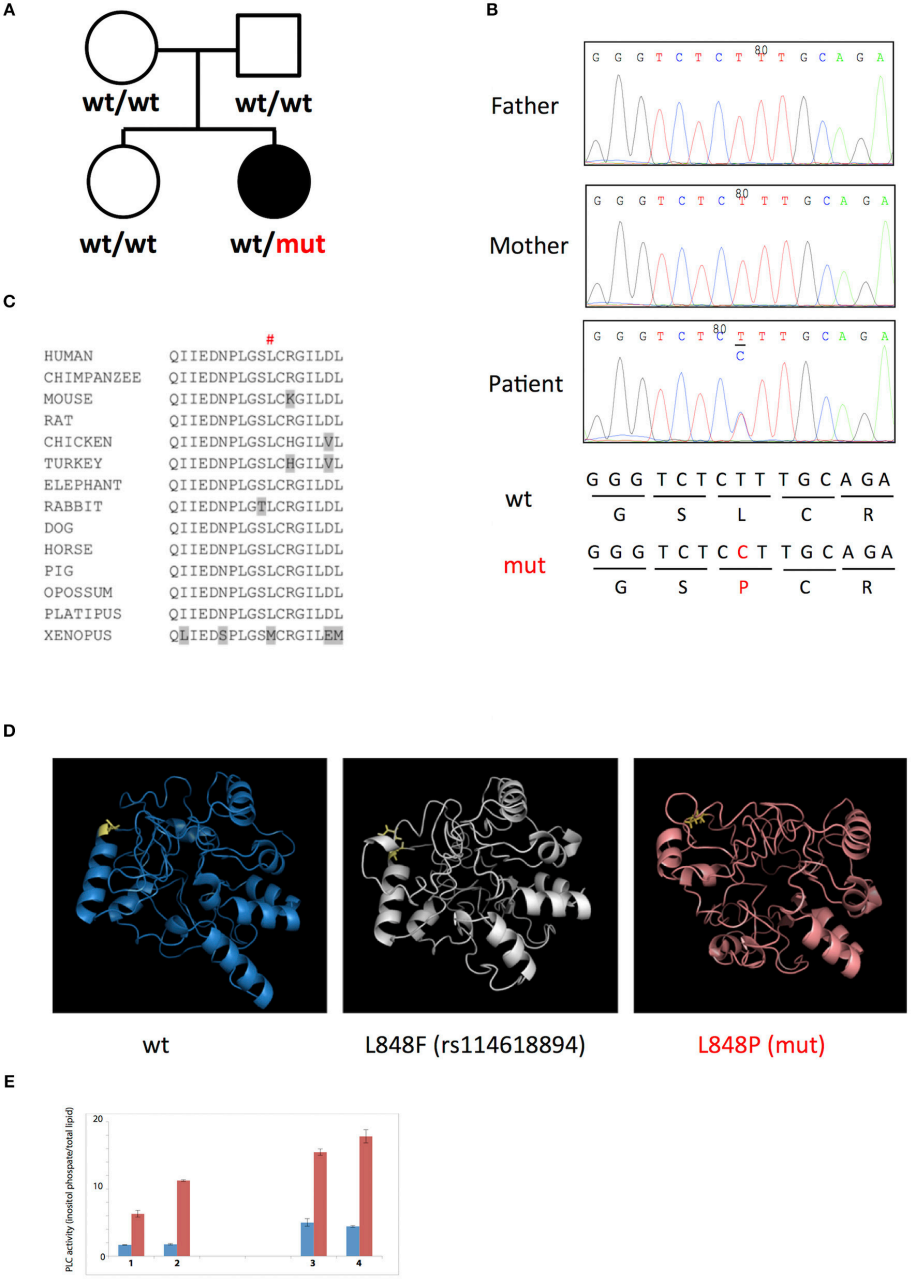
**(A,B)** *De novo* L848P mutation in the PLCG2 gene; **(C)** Leu848 is conserved in most vertebrates; **(D)** L848P mutation is predicted to affect the PLCγ2 structure while L848F had only minimal impact; **(E)** Both L848P (3) and S707Y (4) mutants showed increased basal and EGF-stimulated activity in comparison to the wild-type PLCγ2 (2) and control COS cells (1).

To uncover genetic basis of the disease, we used whole exome sequencing and identified a heterozygous missense L848P mutation in the *PLCG2* gene. Sanger sequencing showed that this mutation appeared *de novo* in the patient, while being absent from both parents and a healthy sibling (Figures 3A,B). The mutation affected amino acid residue Leu848 in the split pleckstrin homology (spPH) domain of the PLCγ2 protein that is conserved in most vertebrates (Figure 3C). The L848P mutation is unique to this patient: it was never found in humans, e.g., it is absent from more than 120,000 subjects in the gnomAD database (12). Interestingly, DNA polymorphism rs114618894 that has ~2% frequency in Africans affects the same amino acid residue leading to the L848F substitution. Bioinformatics analysis predicted that the patient's mutation L848P was damaging (e.g., SIFT = 0.99, MutationTaster = 0.99), while L848F was neutral. We used I-Tasser (17) to model the PLCγ2 protein fragment between amino acids 770 and 1044 containing the L848P and L848F substitutions and found that L848P is predicted to affect the PLCγ2 structure, while L848F had only minimal impact (Figure 3D). The spPH domain contributes to auto-inhibition and also binds Rac. A mutation in this domain has been shown to cause gain of function leading to severe auto-inflammatory disease in mice (13). Furthermore, an activating spPH mutation L845F, which is close to residue L848, has been described in cancer resistance to Ibrutinib, the drug acting on Btk upstream of PLCγ2 (14). In order to test the functional effect of the newly found L848P mutation, we cloned PLCγ2 and introduced by site directed mutagenesis L848P or the previously reported APLAID mutation S707Y. We transfected these constructs into COS-7 cells and measured IP production. In this standard cellular assay, both L848P and S707Y mutants showed increased basal and EGF-stimulated activity in comparison to wild-type PLCγ2 (Figure 3E). Taken together, our data show that L848P is a *de novo* gain-of-function mutation that is very likely to have caused the disease in our patient.

## Discussion

Here, we describe a novel patient with the APLAID syndrome caused by a new missense PLCγ2-activating mutation. This is only the third reported APLAID patient. The clinical and immunological manifestations of our patient resembled the phenotype of the other two APLAID patients described in a single, previous study (11) with the additional presentation of sensorineural deafness and cutis laxa (Figure 1C, Table 2). Interestingly, cutis laxa was also present in two recently identified Spanish patients with APLAID (18) which suggests that it may be frequent among APLAID patients. The *Plcg2*^Ali5^ mice, with a gain-of-function missense mutation in the PLCG2 gene, present inflammatory dermatitis (19). This, and the fact that PLCγ2 is expressed primarily in hematopoietic cells, makes us speculate that this new manifestation is probably driven by persistent skin inflammation.

**Table 2:** Clinical and immunological manifestations of PLAID patients, previously published APLAID patients and this patient.

	**PLAID (*n* = 27)**	**APLAID S707Y (*n* = 2)**	**APLAID L848P (*n* = 1)**
Genetics	heterozygous in-frame deletions PLCG2 gene	Missense mutation	Missense mutation
Mutation effect	Gain-of-function	Gain-of-function	Gain-of-function
PLCγ2 activation status	Constitutive activation	Requires upstream signaling for activation	Requires upstream signaling for activation
Affected PLCγ2 domain	cSH2	cSH2	spPH
**CLINICAL MANIFESTATIONS**			
Cold urticária	✓	✗	✗
Allergic disease	✓	✗	✗
Autoimmunity	✓	✗	✗
Recurrent chest infection	✓	✓	✓
Cutaneous granulomas	✓	✓	✓
Inflammatory bowel disease	✗	✓	✓
Posterior uveitis	✗	✓	✓
Interstitial pneumonitis	✗	✓	✓
Cutis laxa	✗	✗	✓
Sensorineural deafness	✗	✗	✓
**IMMUNOLOGY**			
T cells	Normal	Normal	Normal
Class-switched memory B cells	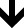	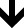	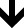
NK cells	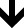	Normal	Normal
IgG	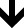	Normal	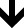
IgA	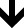	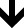	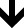
IgM	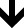	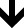	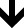
Circulating auto antibodies	✓	✗	✗

On the other hand, despite the fact that congenital and peri-natal causes of sensorineural deafness (infection, malformation, hypoxia) were absent, the patient had received ototoxic medication (such as vinblastine), so it is difficult to define causality.

Recently, Chae et al. (20) found that PBMCs of APLAID patients secreted IL-1β after LPS stimulation, while PBMCs of controls did not, and suggested that systemic inflammation in APLAID is driven by increased activation of the NLRP3 inflammasome (20). Despite this, the two APLAID patients did not respond to IL1 blockers (21). In contrast, we report that levels of IL-1β in whole blood of our patient were decreased after LPS stimulation (Figure 2). The inefficacy of IL1 blockers in our patient also argues against the IL-1-driven mechanism of her disease.

Interestingly, we noticed that levels of IL-10 were substantially less in whole blood of our patient than in controls. IL-10 is an immunomodulatory cytokine that is well known to balance immune response in the gut and impaired signaling in the IL-10 pathway has been associated with IBD (22). Given that all APLAID patients have IBD, it is tempting to speculate that impaired IL-10 production contributes to the immune dysregulation and is responsible for gut inflammation in these patients.

In summary, our report shows that a novel heterozygous missense gain-of-function L848P mutation the *PLCG2* gene causes APLAID in a patient who additionally presented cutis laxa which appears to extend the APLAID phenotype.

## Author Contributions

JN designed the study and wrote the paper. RD designed, performed, and analyzed the cytokine assays. CM, DK, and LB performed immunological analysis. GB-M performed and analyzed the cytokine assays. OP, VP, and JC performed genetic analysis. MM and MK performed the functional validation of the PLCG2 mutation. AC and CN medical doctor of the patient + designed the study. SN designed the study and analyzed the data.

### Conflict of Interest Statement

The authors declare that the research was conducted in the absence of any commercial or financial relationships that could be construed as a potential conflict of interest.
