# Supplementary material for: Novel PLCG2 Mutation in a Patient With APLAID and Cutis Laxa
Source: Front Immunol. 2018 Dec 14;9:2863. doi: 10.3389/fimmu.2018.02863 (PMC6302768; doi:10.3389/fimmu.2018.02863)
Supplement: Supplementary file 1 [file Table_1.docx]

| Stimulus | TNFa | | IL-6 | |
| --- | --- | --- | --- | --- |
|  | **Control** | **Patient** | **Control** | **Patient** |
| Med | 1 | 1 | 0,3 | 0,9 |
| PHA | 2813,4 | 41,8 | 6582,9 | 18,1 |
| PMA+Iono | 17011 | 872,9 | 1962,0 | 156,2 |
| LPS | 2538,9 | 1320,3 | 12440,5 | 10890,5 |
| LPS/IFNg | 8162 | 6804 |  |  |
| PAM2 | 78,9 | 82,9 | 4077,1 | 1068,6 |
| Serum | 0 | 0 | 2,5 | 1,4 |
| PAM3 | 151,3 | 129 | 5969,3 | 2327,3 |
| Flagel | 26,3 | 62,4 | 2118,4 | 1610,4 |
| CL-097 | 2113,1 | 1387,9 | 7403,7 | 4797,1 |

Table E1: Production of IL-6 and TNFα in response to most TLR agonists tested was normal, with the exception of reduced response to ssRNA (agonist of TLR8).
