# Supplementary figures and images for: Novel PLCG2 Mutation in a Patient With APLAID and Cutis Laxa
Source: Front Immunol. 2018 Dec 14;9:2863. doi: 10.3389/fimmu.2018.02863 (PMC6302768; doi:10.3389/fimmu.2018.02863)

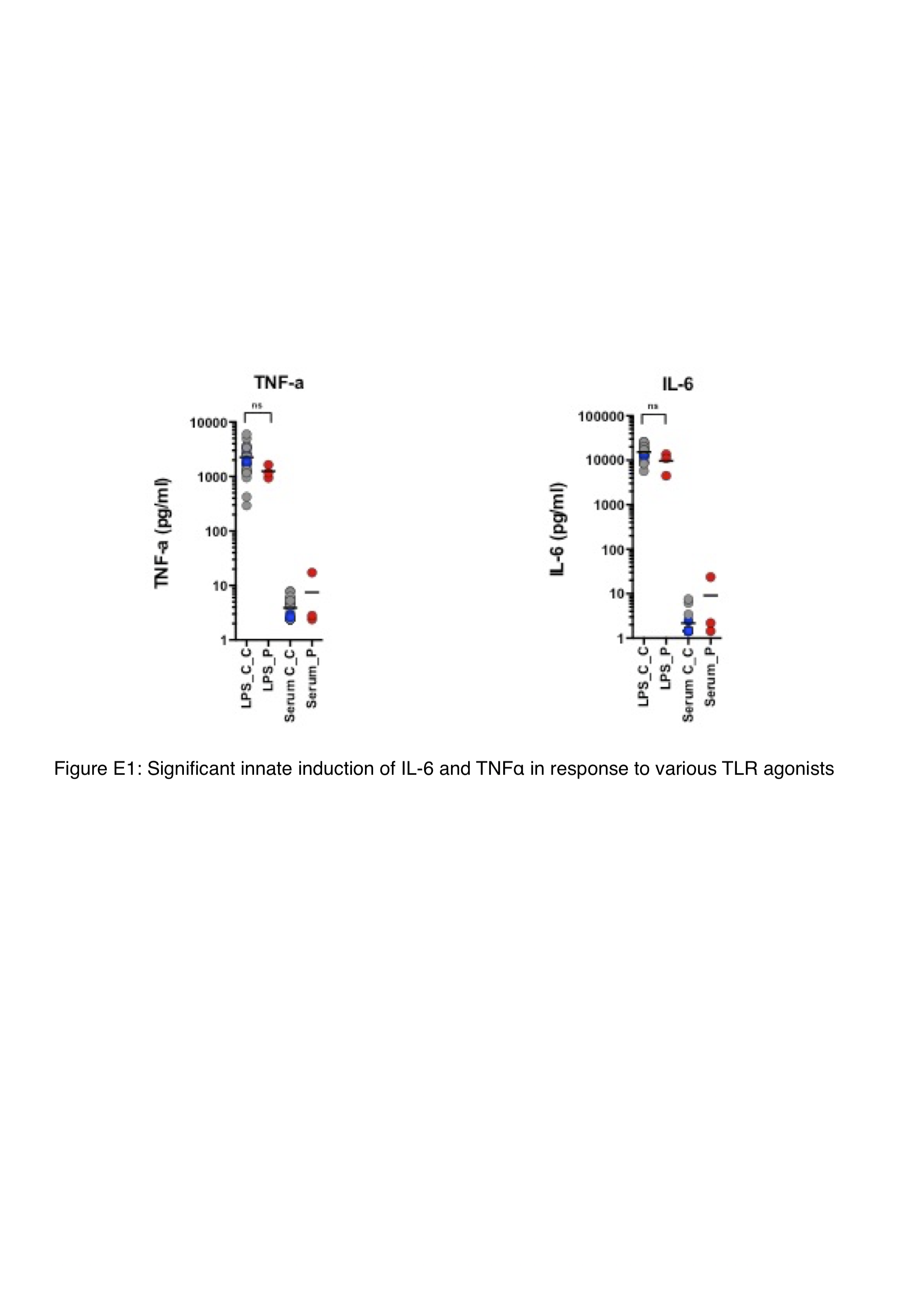

Supplement: Supplementary file 2 [file Image_1.tiff]
